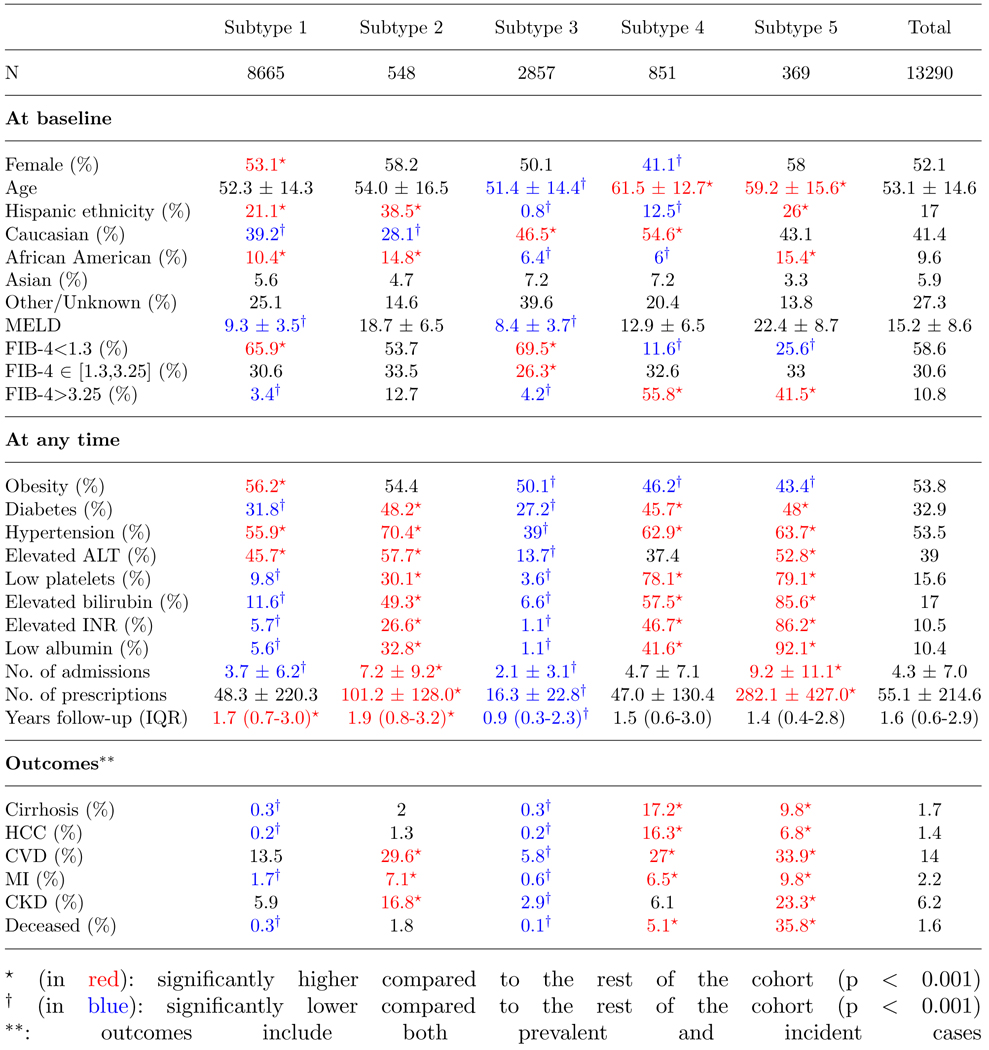# Automated phenotyping of patients with non-alcoholic fatty liver disease reveals clinically relevant disease subtypes

**Published:** 2020

**Authors:** Maxence Vandromme, Tomi Jun, Ponni Perumalswami, Joel T. Dudley, Andrea Branch, Li Li

**Affiliations:** 1 Division of Liver Diseases, Icahn School of Medicine at Mount Sinai, New York, NY 10029, USA; 2 Division of Hematology and Medical Oncology, Icahn School of Medicine at Mount Sinai, New York, NY 10029, USA; 3 Institute for Next Generation Healthcare, Department of Genetics and Genomic Sciences, Icahn School of Medicine at Mount Sinai, New York, NY 10029, USA; 4 Sema4, a Mount Sinai Venture, Stamford, CT 06902, USA

**Keywords:** clustering, subtypes definition, survival analysis, NAFLD

## Abstract

Non-alcoholic fatty liver disease (NAFLD) is a complex heterogeneous disease which affects more than 20% of the population worldwide. Some subtypes of NAFLD have been clinically identified using hypothesis-driven methods. In this study, we used data mining techniques to search for subtypes in an unbiased fashion. Using electronic signatures of the disease, we identified a cohort of 13,290 patients with NAFLD from a hospital database. We gathered clinical data from multiple sources and applied unsupervised clustering to identify five subtypes among this cohort. Descriptive statistics and survival analysis showed that the subtypes were clinically distinct and were associated with different rates of death, cirrhosis, hepatocellular carcinoma, chronic kidney disease, cardiovascular disease, and myocardial infarction. Novel disease subtypes identified in this manner could be used to risk-stratify patients and guide management.

## 1. Introduction

Non-alcoholic fatty liver disease (NAFLD) is estimated to affect 25% of the global population.^1^ NAFLD is a chronic liver disease associated with the metabolic syndrome that can progress to cirrhosis and hepatocellular carcinoma (HCC). In the United States, NAFLD-related liver failure has become the second most common indication for liver transplants, after chronic hepatitis C.^2,3^ This trend is expected to continue, with NAFLD prevalence rising to 33.5% of the adult US population by 2030, and driving increases in both cirrhosis and HCC.^4^

NAFLD is a heterogeneous disease which has been associated with a variety of adverse outcomes. Besides cirrhosis and HCC, NAFLD has also been associated with cardiovascular disease (CVD)^5,6^ and chronic kidney disease (CKD).^7^ In some cohorts, CVD is the leading cause of death among NAFLD patients, followed by malignancy and liver-related mortality.^8–10^

Some NAFLD subtypes and prognostic factors have been identified. Patients with both steatosis and inflammation (i.e. nonalcoholic steatohepatitis, NASH) have worse outcomes than those with bland steatosis.^11,12^ Similarly, patients with NAFLD-associated cirrhosis have worse outcomes than those who do not.^8^ Interestingly, although cirrhosis strongly predicts HCC, some NAFLD patients develop HCC in the absence of cirrhosis.^13^ Hispanic populations tend to have higher rates of NAFLD;^14^ a variant in *PNPLA3* associated with hepatic steatosis and NASH has been identified and is more common among Hispanic individuals.^15^

Given the clinical variability among NAFLD patients, we hypothesized that there may be clinically relevant patient subtypes which could be identified using unbiased machine learning algorithms. The identification of such subtypes could enable more precise prognostication and management for NAFLD patients.

## 2. Methods

### 2.1. NAFLD definition

In order to define NAFLD, we developed an algorithm based on two published electronic medical record (EMR)-based algorithms.^16,17^ First, we identified patients with liver disease based on persistent ALT elevation or ICD codes for chronic non-specific or non-alcoholic liver disease (ICD-9: 571.5, 571.8, 571.9; ICD-10: K75.81, K76.0, K76.9). Persistent ALT elevation was defined as two or more instances of ALT ≥ 40 IU/mL for men, or ≥ 31 IU/mL for women in the ambulatory setting, more than 6 months apart. Then, we excluded patients with viral hepatitis, alcoholic liver disease, or other chronic liver disease. These conditions were identified via ICD codes, as enumerated in the eMerge algorithm. Viral hepatitis cases were also identified using lab values (HBV surface antigen, HCV RNA). Next, we excluded patients on steatogenic medications (defined in eMerge). Finally, patients must have had evidence of hepatic steatosis on imaging, biopsy, or documented in a clinical note. These instances were identified using natural language processing (NLP) to identify mentions of hepatic steatosis and related terms.

### 2.2. Natural language processing

The eMerge algorithm requires mention of hepatic steatosis in a free-form text document (imagery or biopsy result, or clinical note). We developed a tool to get this information from the database, using the following steps:

- build a list of synonyms for the term of interest, e.g. *steatohepatitis*, *fatty liver*
- query the SQL database for documents containing any of these terms
- parse the documents to remove negative results (e.g. *absence of steatohepatitis*), occurrences in family and other false positive patterns

This process was adapted to look for mentions of deceased patients (see Section 2.4), to find patients with cirrhosis (see Section 2.6), and to gather MELD scores (see Table 1).

### 2.3. Data collection

The cohort for this study was created using the criteria defined in Section 2.1. These EMR data were obtained from the database of a large metropolitan hospital in New York City. We choose to only consider patients who met the criteria for NAFLD after December 31, 2012, up to January 31, 2019. We called *NAFLD diagnosis date* the earliest such date for each patient.

13,290 patients matching these criteria were found in the database. In the rest of this section, we describe, for different types of information, the data collection and pre-processing steps that were taken. In order to build a dataset usable by machine learning algorithms, we transformed the information contained in the database into binary features. When possible, we reduced the number of resulting features. Feature selection has been shown to improve the quality of results in machine learning applications.^18^ This process is usually done using statistics- or heuristics-based algorithms. However, in the case of practical applications, we can use domain knowledge instead. We took advantage of established knowledge to reduce the number of features by mapping to higher-level concepts, or discarding infrequent features.

### 2.4. Clinical feature standardization and quality control

#### 2.4.1. Demographic data

- Age: ten mutually exclusive binary attributes corresponding to the following age groups:
  [18–20],[21–30],[31–40],[41–50],[51–60],[61–70],[71–80],[81–90],[91–100],[101 and more].
- Race: Asian, Black, Indian/Native, Pacific Islander, White, Hispanic, Other, Unknown
- Ethnicity: Hispanic or not
- Deceased: obtained through patient records and parsing clinical notes for mentions of death

#### 2.4.2. Diagnoses, procedures, medications

A large proportion of clinical data overall can be described through standardized coding systems: diagnoses, procedures, medications. We applied the following preprocessing steps:

- Diagnoses used the International Classification of Diseases, versions 9 and 10 (ICD-9 and ICD-10) systems. These systems contain a tens of thousands of different codes, often describing the same disease with minor variations. In order to reduce the number of features, we used the *phecode* system from the Phenome Wide Association Studies (PheWAS).^19^ We kept only phecodes with at least 0.1% prevalence, which left 148 features for ICD codes.
- Procedures used the Current Procedural Terminology (CPT) coding system. We mapped the CPT codes to their respective second-level group code. For example, the group containing all CPT codes from 33010 to 37799 describes surgeries of the cardiovascular system. This process grouped the codes into 115 categories that translated directly into features.
- Medication prescriptions or administrations. We mapped the medication names to the corresponding RxNorm drug concepts, and again kept those that occurred in at least 0.1% of the cohort. We only considered drugs which had at least two prescriptions separated by 6 months or more, in order to discard drugs only used acutely (e.g. post-surgery) which do not reflect a patient’s regular medications. Using this process, we obtained 293 clinical drugs.

#### 2.4.3. Laboratory tests

As opposed to the previous data types, which were well-formatted and standardized, laboratory tests could be either qualitative or quantitative, and were often reported in free-text form. For qualitative tests, we parsed the result and searched for terms that indicated if it was abnormal, such as *abnormal*, *low*, *below average*, *reactive*. For quantitative tests, we searched the results for numeric values that fell outside the normal range.

We obtained 533 distinct laboratory tests, which translated to as many binary features. For example, feature *platelets* means *abnormal result for platelets test*. A shortcoming of this approach is that abnormally low and high values are grouped in the same feature, even though they have different medical significance. However, since one laboratory test can use different units, and thus different normal ranges (e.g. normal and log scales), automatically assigning a value to *low* or *high* is not always reliably doable.

#### 2.4.4. Vital signs

Similar to laboratory tests, we searched for abnormal values for the standard vital signs collected in clinical settings, using the following criteria:

- body temperature: > 39°C (Celsius) or 102^◦^F (Fahrenheit).
- blood pressure: systolic/diastolic blood pressure (SBP/DBP) > 130/80
- heart rate: > 130 bpm.
- respiratory rate: > 40 bpm.
- pain: values of 9 or 10 on a [1–10] pain scale.

### 2.5. Patient pairwise distance and clustering

In order to identify different subtypes, we computed the patient distance matrix and applied an algorithm of unsupervised clustering to the data obtained. Unsupervised clustering is well-suited for exploratory tasks in applied research.^20^ First, validation of the results obtained using expert knowledge is possible. In the present study, the findings were reviewed and interpreted by medical experts. Second, the “unsupervised” aspect allows discovery of new, potentially unexpected insight from the analysis of a large number of features.

Many clustering algorithms have been developed. Finding the “best one” remains an open problem,^21^ since unsupervised learning tasks lack objective measures to assess their performance. Several measures have been proposed to evaluate the quality of a set of clusters,^22^ but the general guideline is that the best algorithm and parameters are different for each data set.

We chose a hierarchical clustering algorithm using the Manhattan distance for pairwise similarity of patients, and minimizing the increase in variance during cluster merging as linkage criterion (also known as Ward’s criterion). Hierarchical clustering is a standard algorithm, and it has been used previously in a study looking for comorbidity clusters in autism disorders.^23^ We used the R *hclust* implementation of this algorithm, with *ward.D2* as parameter for agglomeration criterion.^24^ We chose to have 5 subtypes (clusters) as a balance between granularity and size. These parameters were chosen empirically, after qualitative validation of the results obtained with various combinations.

### 2.6. Statistical analysis

#### 2.6.1. Descriptive statistics

Categorical features were summarized as proportions and compared using the chi-squared test. Continuous features were summarized as means ± standard deviation and compared using ANOVA, or as medians and interquartile ranges compared using the Wilcoxon rank-sum test. Comparisons for each subtype were made against patients in all remaining subtypes. Significance was defined as a false discovery rate *<*0.001.

#### 2.6.2. Survival analysis

The primary outcome was overall survival. Secondary outcomes were HCC, cirrhosis, CKD, CVD, and acute myocardial infarction (MI). In all cases survival was defined as the time from NAFLD diagnosis to the earliest evidence of the outcome. HCC cases were first identified using ICD codes (ICD-9 155.0,155.2; ICD-10 C22.0,C22.7-C22.9), then confirmed through chart review. Cirrhosis was defined using natural language processing looking for mentions of cirrhosis in clinical notes, imaging reports or biopsy reports. Chronic kidney disease was defined using corresponding ICD codes (ICD-9 585–586; ICD-10 N18-N19) and CPT codes for dialysis (90935 to 90999). Cardiovascular disease was defined using ICD codes for any ischemic heart disease (ICD-9 410–414; ICD-10 I20-I25). Acute MI was a subset of the CVD outcome (ICD-9 410; ICD-10 I21-I22).

The primary predictor in survival analyses was subtype. Secondary predictors included age, gender, race and FIB-4 category. Race and ethnicity were combined for the purposes of this analysis, with Hispanic ethnicity given precedence and mapped to the Hispanic race category. The primary outcome was overall survival. Secondary outcomes were onset of cirrhosis, HCC, CVD, MI, and CKD. All survival analyses were done in R 3.6.0. For the outcome of overall survival, Kaplan-Meier curves were created using the *ggplot2*^25^ and *survminer*^26^ packages; univariate and multivariate Cox proportional hazards models were constructed using the *survival* package.^27^ For non-death outcomes, only incident cases were included in the analysis. Cases diagnosed prior to or within 6 months of NAFLD diagnosis were treated as prevalent. Death was treated as competing hazard. The cumulative incidence function was calculated for each outcome using the *cmprsk* package^28^ and plotted using *ggplot2*. The *cmprsk* package was also used to fit univariate and multivariate Fine-Gray proportional subdistribution hazards regression models for the non-death outcomes.

This study was reviewed and approved by the Mount Sinai Hospital institutional review board (GCO 10–0032 and 16–1437).

## 3. Results

### 3.1. Descriptive statistics for the cohort

Merging the data from the different sources described above, we obtained a data set containing 13,290 patients with NAFLD, described by 1,145 binary features (Table 1). The mean age at NAFLD diagnosis is 53 ± 14.7 (median = 53.9), with 50.6% female patients. The cohort was racially and ethnically diverse: 41.4% Caucasian, 17% Hispanic ethnicity, 9.6% African American, 5.9% Asian, and 27.3% unknown/other. Metabolic comorbidities such as obesity (53.8%), diabetes (32.9%), and hypertension (53.5%) were common. Median length of follow up was 1.6 years (IQR 0.6–2.9).

### 3.2. Identification of NAFLD subtypes

The two largest subtypes (1 and 3) encompassed 87% of patients, while the remaining patients are divided among 3 smaller subtypes (Table 1). All findings reported below were for the comparison of subtype members versus all other patients, and were significant after correction for multiple hypothesis testing at a level of p*<*0.001. Values associated with medications are omitted for concision.

Patients in subtype 1 were more likely to be female and either Hispanic or African American. Obesity, hypertension, and hyperlipidemia (30.05 vs 24.8%) were more common among subtype 1 patients, while diabetes was less common. Subtype 1 patients had low MELD and FIB-4 scores at NAFLD diagnosis. Other diagnoses more common in subtype 1 patients included: vitamin D deficiency (14.2% vs 9.2%), asthma (11.4 vs 7.5%), gastroesophageal reflux (18.7% vs 12.7%). Medications that were more common in this subtype included: omeprazole, metformin, atorvastatin, and fluticasone. Overall, subtype 1 patients had metabolic comorbidities, with some evidence of liver inflammation, but minimal liver fibrosis.

Patients in subtype 2 were more likely to be Hispanic or African American. They did not have significantly higher MELD or FIB-4 scores at baseline, but they were more likely than other patients to have labs suggestive of liver inflammation and dysfunction, such as elevated ALT, low platelets, elevated bilirubin, elevated INR and low albumin. Notable comorbidities included: diabetes, hypertension, hyperlipidemia (37.2% vs 27.8%), obstructive sleep apnea (11.9% vs 6.0%), gastroesophageal reflux (27.2% vs 16.1%), tobacco use (19.5% vs 4.8%), asthma (22.1 vs 9.5%), anxiety (13.0% vs 5.6%), depression (17.0% vs 6.8%), urinary tract infection (11.5% vs 3.9%), and respiratory infection (10.6% vs 3.6%). Medications more commonly prescribed in this subtype included cardiac medications such as aspirin, lisinopril, amlodipine, metoprolol, and atorvastatin; diabetes medications such as metformin and insulin; pain medications such as acetaminophen, gabapentin, oxycodone, and morphine; respiratory medications such as albuterol and fluticasone; antacid medications such as omeprazole and famotidine, and also vitamin D. Subtype 2 patients were also more likely to have had digestive surgery (40.1% vs. 16.8%). Overall, subtype 2 patients had metabolic syndrome with signs of developing liver dysfunction and were high healthcare utilizers.

Patients in subtype 3 tended to be younger, Caucasian and had the fewest inpatient admissions and the fewest prescriptions on average. Subtype 3 patients had fewer comorbidities than other patients, and were unlikely to have abnormal lab values associated with liver dysfunction. Subtype 3 patients were relatively healthy compared to the rest of the cohort.

Patients in subtype 4 were more likely to be older, male and Caucasian. They had high FIB-4 scores at baseline and were likely to have abnormal labs suggesting liver synthetic dysfunction. These patients were less likely to be obese or to have hyperlipidemia (20.8% vs 28.7%), though diabetes and hypertension were common. Overall, subtype 4 patients likely had liver fibrosis at baseline and had labs suggesting progression to cirrhosis.

Patients in subtype 5 were more likely to be older, and Hispanic or African American. They had high FIB-4 and MELD scores at baseline, and had high rates of abnormal lab values consistent with liver inflammation and dysfunction. Obesity was less common in this group, but diabetes and hypertension were prevalent. Other comorbidities included: malignancy (15.2% vs 2.0%), atrial fibrillation (11.4% vs 1.6%), tobacco use (28.7% vs 4.7%), depression (17.1% vs 6.9%), urinary tract infection (16.8% vs 3.8%), pneumonia (10.3% vs 1.9%), and sepsis (25.2% vs 0.3%). Commonly prescribed medications included: cardiac medications such as aspirin, metoprolol, and furosemide; pain medications such as acetaminophen, oxycodone, hydromorphone, fentanyl, and morphine; antacid medications such as pantoprazole and famotidine; and insulin. Subtype 5 patients were also more likely to have had cardiovascular (31.4% vs 7.4%), respiratory (16.5% vs 4.6%) or digestive surgery (50.0% vs 16.9%). Overall, subtype 5 patients had significant liver disease at baseline, had significant cardiac, infectious and neoplastic comorbidities, and were high healthcare utilizers.

### 3.3. Identification of distinct outcomes by NAFLD subtype

**Fig. 1.**
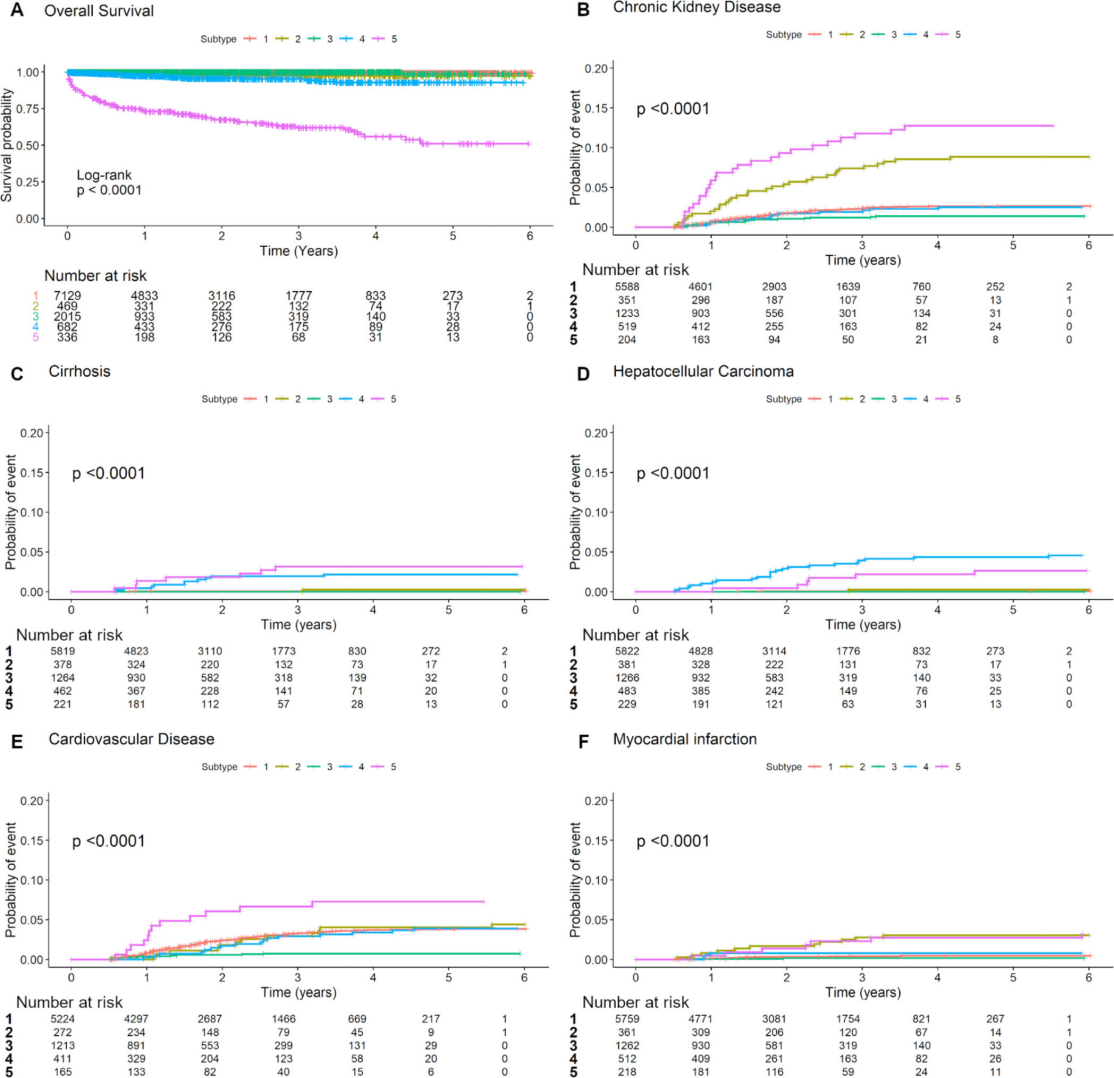
Survival and hazard curves for outcomes of interest, 5 by subtypes. (A) Overall survival, (B) Chronic kidney disease, (C) Cirrhosis, (D) Hepatocellular carcinoma, (E) Cardiovascular disease, (F) Myocardial infarction.

**Fig. 2.**
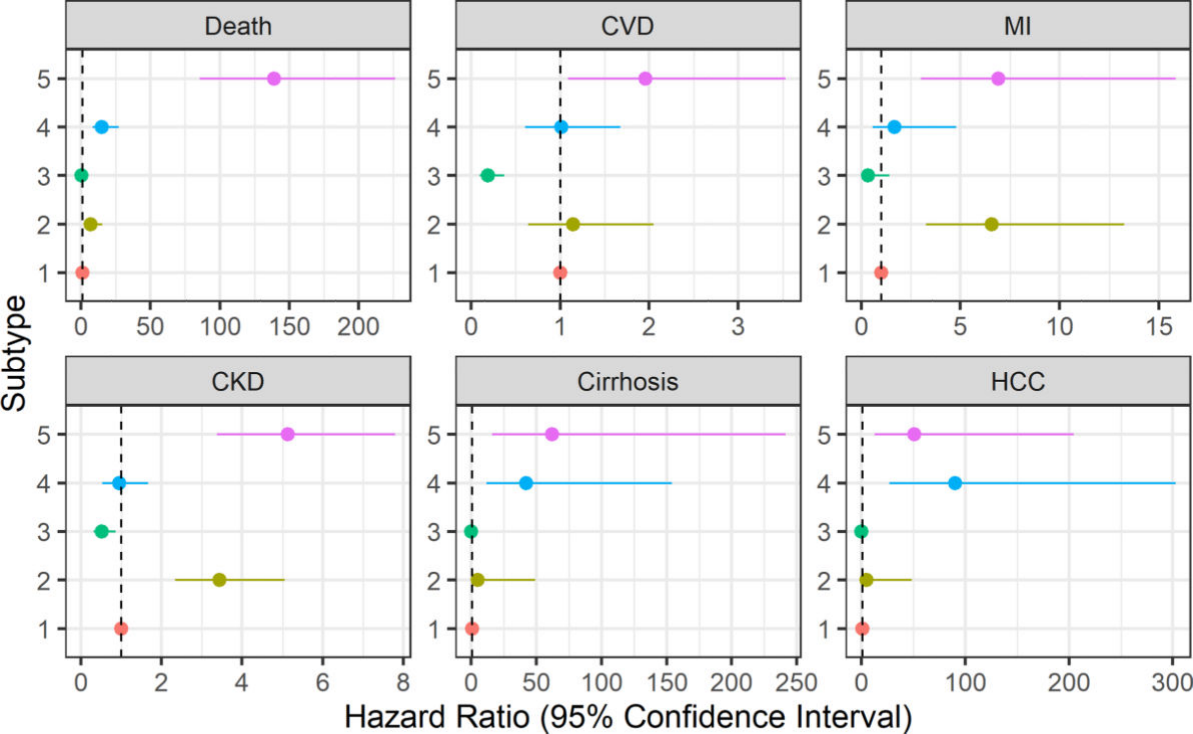
Univariate hazard ratios for outcomes of interest, by 5 subtypes

Univariate analyses showed that risk of outcomes varied by subtype membership (Figures 1 and 2). Subtype 1 was chosen as the reference group since it was the largest. Compared to subtype 1, subtype 5 was significantly and strongly associated with an increased risk of all outcomes; risk of death was particularly high (HR 139; 95% CI 86–226, p*<*0.001). Subtype 4 was strongly associated with both cirrhosis (HR 42; 95% CI 12–154, p*<*0.001) and HCC (HR 91; 95% CI 27–302, p*<*0.001). Subtype 2 was associated with MI (HR 6.6; 95% CI 3.3–13.3, p*<*0.001) and CKD (HR 3.4; 95% CI 2.3–5.1, p*<*0.001). Subtype 3 was associated with a lower risk of CVD (HR 0.19; 95% CI 0.10–0.37, p*<*0.001), and CKD (HR 0.51; 95% CI 0.31–0.86, p=0.01). There were no incident cirrhosis or HCC events in group 3.

**Fig. 3.**
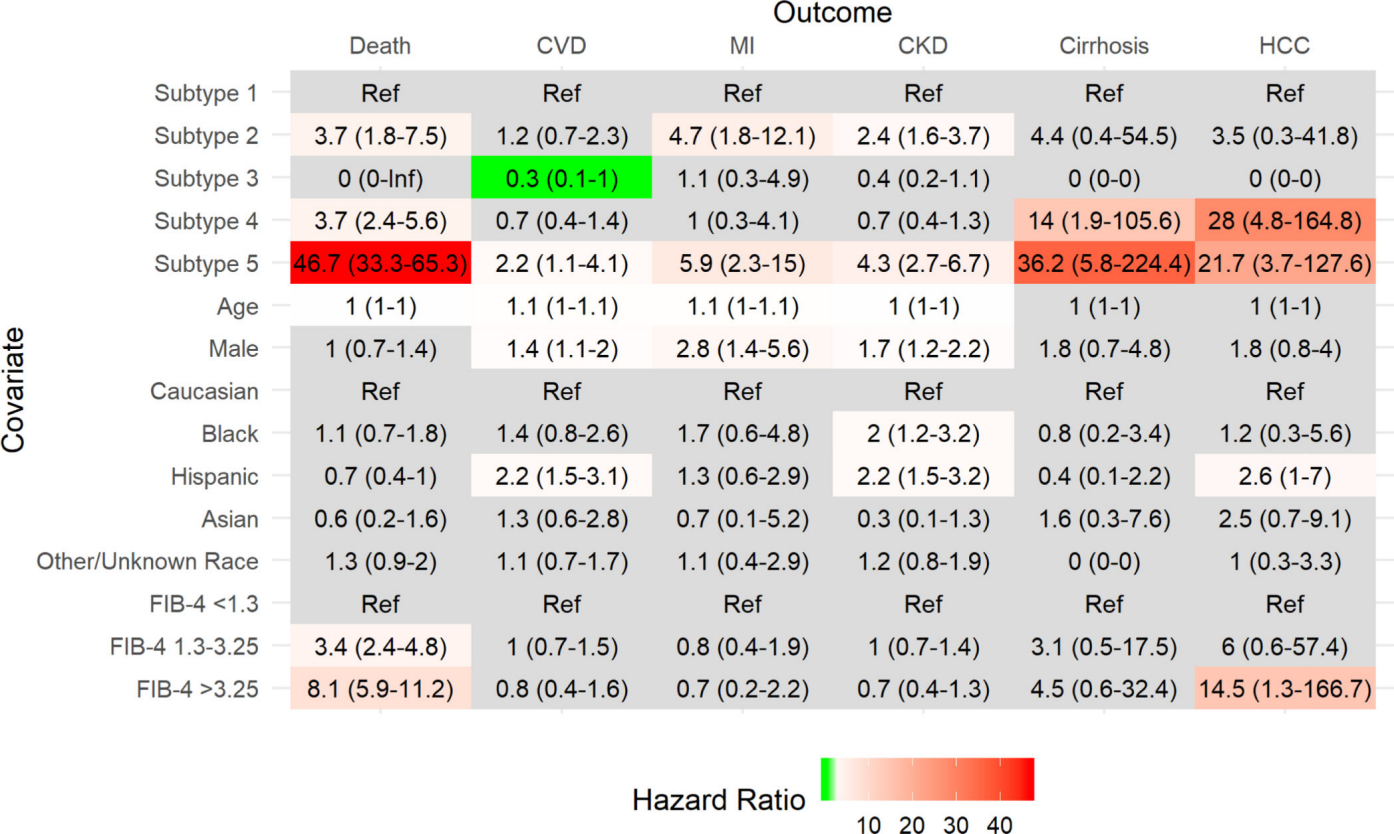
Multivariate analyses for outcomes of interest. Darker shades of red correlate with increased risk of the outcome, while darker shades of green indicate reduced risk of the outcome. Only hazard ratios with p*<*0.05 are color coded. Non-significant findings are in grey.

In multivariate analyses accounting for age, gender, race and baseline FIB-4, subtype membership remained an independent predictor of outcomes (Figure 3). With subtype 1 as the reference, Subtype 5 was independently associated with the highest risks for death (HR 46.7; 95% CI 33.3–65.3, p*<*0.001), CKD (HR 4.3; 95% CI 2.7–6.7, p*<*0.001), CVD (HR 2.2; 95% CI 1.1–4.1, p=0.02 ), MI (HR 5.9; 95% CI 2.3–15.0, p*<*0.001) and cirrhosis (HR 36.2; 95% CI 5.8–224.4, p*<*0.001) among all subtypes, while subtype 4 was independently associated with a high risk for cirrhosis (HR 14.0; 95% CI 1.9–105.6, p=0.01) and the highest risk for HCC (HR 28.0; 95% CI 4.8–164.8, p*<*0.001). Subtype 2 was also independently associated with an elevated risk of death (HR3.7; 95% CI 2.4–5.6, p*<*0.001), MI (HR 4.7; 95% CI 1.8–12.1, p*<*0.001) and CKD (HR 2.5; 95% CI 1.6–3.7, p*<*0.001). Subtype 2 was the only other subtype aside from subtype 5 to be independently associated with MI and CKD.

### 3.4. Internal cross-validation of the subtypes discovered

Formal validation of the results is inherently complicated for unsupervised clustering, where no “true label” exist for any patient. In order to assess the robustness of our results, we have performed internal cross-validation on our dataset, as we have no access to EMR in other medical centers. We have randomly selected 90% of samples, run the clustering process on this new training set, and repeated the process 10 times. We have identified similar enriched clinical features and disease comorbidities in the subtypes that we have discovered previously. We reported the full results in the supplementary table 1 hosted at https://github.com/mv50/psb20_mat.

## 4. Conclusion

In this study, we combined two existing signatures of NAFLD and used them to gather a cohort of 13,290 patients with confirmed NAFLD. We used unsupervised clustering to identify five subtypes of patients. These subtypes had different clinical characteristics and different outcomes: the two larger groups had fewer comorbidities and more positive outcomes, while a minority of the cohort (in the three smaller subtypes) had more serious comorbidities and worse outcomes. To our knowledge, this study is the first to use an artificial intelligence approach to delineate clinically relevant subtypes of NAFLD.

Our findings are consistent with prior studies reporting higher rates of NAFLD among Hispanic patients.^14^ In addition, the subtypes reveal that Hispanic patients with NAFLD are on a continuum of risk, with some exhibiting the metabolic syndrome but having good outcomes (subtype 1), others experiencing predominantly non-liver adverse outcomes (subtype 2) and some with severe liver disease and at risk for multiple adverse outcomes (subtype 5).

Our study of heterogeneity among NAFLD patients was strengthened by the diverse patient population within Mount Sinai’s catchment area and the comprehensive use of EMR records. We gathered data from various sources to build the features: vital signs, diagnoses, procedures, prescriptions, laboratory results, radiology and pathology reports. Our approach is generalizable and could be applied by local or regional healthcare systems to define disease subtypes within their own patient populations. Such efforts could help guide resource allocation at the local level, in contrast to national or international guidelines which may not be relevant to all localities and patient populations.

The limitations of our study are common to EMR-based projects. ICD codes are prone to miscoding and may not accurately represent a patient’s medical condition. We used phecodes to map ICD codes to higher-level disease concepts in order to improve power and simplify instances where there are multiple related ICD codes. The pre-processing and cleaning of the data remains open to improvements. Additionally, more systematic incorporation of data from unstructured clinical notes could bring valuable new information.

In conclusion, we defined an EMR-based algorithm for identifying NAFLD patients and showed that unsupervised clustering can be used to identify clinically relevant disease subtypes with distinct patterns of adverse outcomes. If prospectively validated, these disease subtypes could help guide patient management and screening initiatives.

## Supplementary Material

1

## Figures and Tables

**Table 1.** Baseline characteristics, selected features of interest, and outcomes by subtype