# Supplementary material for: Automated phenotyping of patients with non-alcoholic fatty liver disease reveals clinically relevant disease subtypes
Source: Pac Symp Biocomput. Author manuscript; Available in PMC 2020 Feb 26. (PMC7043281)
Supplement: 1 [file NIHMS1061138-supplement-1.pdf]

Supplementary table 1 : comparison of outcomes and main clinical features in the original data ('base') and the cross-validation ('partition') over 10 iterations

Comorbidities	partition_subtype_1	base_subtype_1	partition_subtype_2	base_subtype_2	partition_subtype_3	base_subtype_3	partition_subtype_4	base_subtype_4	partition_subtype_5	base_subtype_5
Cirrhosis	**0.4 +/- 0.1**	**0.3**	**2.5 +/- 3.0**	**2**	**0.3 +/- 0.2**	**0.3**	**15.0 +/- 3.5**	**17.2**	**8.9 +/- 1.9**	**9.8**
HCC	**0.2 +/- 0.1**	**0.2**	**1.6 +/- 2.3**	**1.3**	**0.2 +/- 0.1**	**0.2**	**13.9 +/- 3.3**	**16.3**	**6.0 +/- 1.6**	**6.8**
CVD	11.2 +/- 0.9	13.5	44.6 +/- 11.5	29.6	19.1 +/- 6.9	5.8	**23.7 +/- 3.5**	**27**	**32.0 +/- 3.3**	**33.9**
MI	**1.6 +/- 0.1**	**1.7**	**8.6 +/- 2.6**	**7.1**	2.0 +/- 0.7	0.6	**5.6 +/- 1.2**	**6.5**	**8.8 +/- 1.6**	**9.8**
CKD	4.9 +/- 0.4	5.9	29.8 +/- 8.9	16.8	8.6 +/- 3.7	2.9	**6.7 +/- 1.1**	**6.1**	**20.1 +/- 3.3**	**23.3**
Dead	**0.3 +/- 0.0**	**0.3**	**5.6 +/- 5.5**	**1.8**	**0.2 +/- 0.1**	**0.1**	**5.0 +/- 1.4**	**5.1**	**35.9 +/- 8.8**	**35.8**
Obesity	53.9 +/- 1.1	56.2	**57.1 +/- 6.2**	**54.4**	58.7 +/- 5.4	50.1	**48.0 +/- 2.8**	**46.2**	**44.1 +/- 2.2**	**43.4**
Diabetes	28.4 +/- 1.9	31.8	66.0 +/- 14.1	48.2	52.3 +/- 18.3	27.2	**42.2 +/- 4.8**	**45.7**	**46.5 +/- 5.2**	**48**
Hypertension	50.5 +/- 2.5	55.9	83.8 +/- 13.1	70.4	67.1 +/- 16.1	39	**59.9 +/- 2.9**	**62.9**	**66.0 +/- 3.0**	**63.7**
Elevated ALT	38.2 +/- 4.1	45.7	**60.2 +/- 4.2**	**57.7**	43.4 +/- 16.1	13.7	40.5 +/- 2.0	37.4	**54.2 +/- 3.0**	**52.8**
Low platelets	8.4 +/- 1.0	9.8	**36.5 +/- 13.4**	**30.1**	13.0 +/- 4.3	3.6	**68.1 +/- 12.1**	**78.1**	**76.9 +/- 5.0**	**79.1**
Nb patients	8721 +/- 704	8665	252 +/- 140	548	1709 +/- 720	2857	970 +/- 385	851	309 +/- 118.0	369
